# Trends, Diagnoses, and Hospitalization Costs of Child Abuse and Neglect in the United States of America

**DOI:** 10.3390/ijerph18147585

**Published:** 2021-07-16

**Authors:** Armeda Stevenson Wojciak, Brandon Butcher, Aislinn Conrad, Carol Coohey, Resmiye Oral, Corinne Peek-Asa

**Affiliations:** 1 Department of Family Social Science, College of Education and Human Development, University of Minnesota, St. Paul, MN 55108, USA; 2 Injury Prevention Research Center and Department of Biostatistics, College of Public Health, University of Iowa, Iowa City, IA 52242, USA; brandon-butcher@uiowa.edu (B.B.); corinne-peek-asa@uiowa.edu (C.P.-A.); 3 School of Social Work, University of Iowa, Iowa City, IA 52242, USA; aislinn-conrad@uiowa.edu (A.C.); carol-coohey@uiowa.edu (C.C.); 4 Children’s Hospital at Dartmouth, Geisel School of Pediatrics, Dartmouth College, Hanover, NH 03756, USA; Resmiye.oral@hitchcock.org

**Keywords:** child maltreatment, hospitalization rates, hospitalization costs, National Inpatient Sample

## Abstract

We conducted a secondary analysis of the National Inpatient Sample (NIS) to examine child abuse and neglect hospitalization from 1998–2016. The NIS is the largest all-payer, inpatient care database in the United States and is maintained by the Health Care Utilization Project. Participants were youth 18 years and younger with discharged diagnoses of child abuse and neglect from hospitals. The rate of child abuse or neglect hospitalizations did not vary significantly over the study period (1998–2016), which on average was 6.9 per 100,000 children annually. Males (53.0%), infants (age < 1; 47.3%), and young children (age 1–3; 24.2%) comprised most of the child maltreatment cases. Physical abuse was the most frequent type of maltreatment leading to hospitalization. Government insurance was the most common payer source, accounting for 77.3% of all child maltreatment hospitalizations and costing 1.4 billion dollars from 2001–2016. Hospitalizations due to child abuse and neglect remain steady and are costly, averaging over $116 million per year. The burden on government sources suggests a high potential for return on investment in effective child abuse prevention strategies.

## 1. Introduction

Child maltreatment is a global problem [1,2] with approximately 1 billion youth between ages 2–17 experiencing past-year violence [3]. Child victims of maltreatment experience poorer outcomes, including depression, inadequate physical health, suicidal ideation [4,5,6,7], lifelong health effects and early death [8]. In the United States, approximately 1 in 3 children will have a maltreatment investigation before the age of 18, with 1 in 8 children experiencing abuse or neglect during childhood [9,10]. In addition to consequences for children, child maltreatment is a pervasive public health problem on the rise. Since 2015, maltreatment referrals have increased by 7.2% in the United States [11]. Less is known, however, about trends in child maltreatment hospitalizations or the economic burden of child maltreatment on our US healthcare system. This is problematic because hospitalizations represent severe instances of child maltreatment. In the absence of trend data, researchers cannot determine whether hospitalization trends are similar to trends in child maltreatment investigations, nor develop strategies to mitigate these trends.

### 1.1. Child Maltreatment Hospitalizations Trends in the United States

If trends in hospitalizations mirror trends in maltreatment investigations, we expect an increase in hospitalizations and negative consequences for both children and the healthcare system. An increased number of hospitalizations would indicate that more children are experiencing more severe maltreatment despite concerted prevention efforts through parenting programs [12] and universal violence prevention programs [13]. Though researchers find unchanging rates of national child maltreatment hospitalizations among young children (0–10) across two time periods (1997–2009 Farst [14]; 2006–2014 Zins [15]), it is difficult to compare these findings to national trends in child maltreatment investigations for two reasons. First, it is difficult to summarize findings from available trend studies given differences in measures and types of maltreatment hospitalizations assessed, and time periods and samples (Farst [14] [ages 0–3; NIS sample]; Rebbe [16] [ages 0–3; Washington State]; Zins [15] [ages 0–10; Emergency room and NIS sample]). Second, whereas national trends in maltreatment investigations include children of all ages (0–18), trends in maltreatment hospitalizations apply to young children (0–10), which does not account for the experiences of older children who have increased cumulative risk for experiencing more and varying types of child maltreatment [9].

In response to the limitations described above, we examined trends in child maltreatment hospitalizations among children of all ages (0–18) from 1998 to 2016. Having this data will help researchers determine whether severe forms of abuse have declined or increased per hospitalization trends, which will enable child-serving agency directors to determine whether their prevention efforts have been successful.

### 1.2. Cost of Child Maltreatment Hospitalizations

The economic burden of child maltreatment on our health care system is an important consideration. Brown, Fang, and Florence [13] examined eight studies in their systematic review of medical costs associated with child maltreatment. The per-episode maltreatment hospital costs ranged from $0 to more than $12,557, with the largest costs associated with traumatic brain injuries ($4232 to $24,411). The only nationally representative study in Brown’s [17] systematic review found the total inpatient charges for maltreated children was $19,266 compared to $9513 for children who were not maltreated [18]. The cost disparity was replicated in other studies using national samples [19,20].

National estimates of the total cost of hospital stays associated with child maltreatment are substantial. In 2005, the estimated cost of maltreatment hospitalizations was $98.7 million [20]. There are costs related to specific types of child maltreatment as well, including physical abuse or pediatric head trauma [17,20]. National estimates for hospital stays for physical abuse were $73.8 million annually [19]. Similarly, a five-year estimate of national hospital costs associated with pediatric abusive head trauma totaled $67 million [21]. Additionally, Rovi [18] reported that children hospitalized for maltreatment had double the cost and number of diagnoses than children hospitalized with diagnoses other than maltreatment.

Despite having data on overall maltreatment costs and individual subtypes, few researchers have investigated the costs of various types of maltreatment hospitalizations over time within one study. Rovi, [18] for example, reported that the highest costs of maltreatment hospitalizations were related to shaken baby syndrome (SBS; $30,311), while the lowest costs were related to emotional abuse ($9875).

Overall, research on child maltreatment hospitalizations indicates the costly nature of maltreatment hospitalizations, yet does not describe how hospitalization costs trend over time for overall or individual forms of maltreatment, nor who pays for child maltreatment hospitalizations. This knowledge gap is troubling given the substantial costs associated with hospitalizations. Though we have data on one-year snapshots [18,19,20] or a five-year interval for one type of child maltreatment, [21] less is known about payers for overall or individual maltreatment hospitalization costs over several years. Across studies, a public payer such as Medicare and Medicaid paid most hospital costs (Peterson [21]: 60%; Rovi [18]: 66.5%; Russo [20]: 70.6%). To put the economic burden of child maltreatment into context, Medicaid only covered 37% [18] to 45.6% [20] of all non-child maltreatment hospital costs.

To provide a more comprehensive understanding of trends in child maltreatment hospitalizations and address gaps in the literature, we estimated the rate of child maltreatment over 16 years, the frequency and type of child maltreatment hospitalizations by demographic characteristics, and trends for hospital costs by payer source.

## 2. Materials and Methods

### 2.1. Sample

Children aged 18 years and younger who were discharged following hospital admission with a diagnosis or external cause code indicating abuse or neglect between 1998 through 2016 were included in this retrospective study of the National Inpatient Sample (NIS). The NIS is the largest all-payer, inpatient care database in the US and is maintained by the Health Care Utilization Project [22]. All analyses incorporated NIS annual sampling weights to estimate national trends. See Table 1 for a description of the sample.

### 2.2. Measures

Child abuse or neglect admissions were identified with external cause of injury codes (E-code E967.0–E967.9) and diagnosis codes (995.5 and 995.50–995.59) based on the International Classification of Diseases [23] (ICD-9-CM and ICD-10-CM in Q4 2015–2016). Corresponding ICD-10-CM codes were identified using MapIT20 software developed by the Agency for Healthcare Research and Quality and the Centers for Disease Control and Prevention.

E-codes include information about the abuse perpetrator, which were grouped into six categories: (1) father/step-father/boyfriend (E967.0), (2) mother/step-mother/girlfriend (E967.2), (3) all other relatives (E967.3–E967.7), (4) non-relative (E967.8), (5) multiple (any child with more than one category of the previous E-codes), and (6) not specified/other/none (E967.1, E967.9, or no E-code). Diagnosis codes identified the type of child maltreatment, which were grouped into seven categories: (1) neglect (995.52), (2) physical abuse (995.54), (3) psychological abuse (995.51), (4) shaken baby syndrome (995.55), (5) sexual abuse (995.53), (6) multiple (any child with more than one diagnosis codes), and (7) not specified/other/none (995.50, 995.59, or no diagnosis code). Payer source was categorized as (1) government (e.g., Medicaid), (2) private insurance, (3) self-pay, (4) other.

### 2.3. Analytic Strategy

The annual prevalence of child abuse and neglect hospitalizations was calculated from 1998 through 2016. Hospitalizations were the weighted total estimate of cases identified through E-codes and diagnostic codes. A chi-square test was used to test for a significant effect in the number of cases due to transitioning from ICD-9-CM to ICD-10-CM in Q4 2015. Rates were calculated by dividing hospitalizations by the number of children under age 18 for each year.

One primary question of interest was whether the mean admission-level costs and the annual total hospital-level costs differed over time by payer source. The NIS-provided user guide was followed for converting charges to costs by using the provided cost-to-charge ratio files [24]. Costs were then adjusted for inflation to Q4 of 2016 for inpatient hospital services based on the Bureau of Labor Statistic’s Consumer Price Index. Because cost-to-charge information was only available from 2001 onward, analyses involving costs included 2001–2016 only. Data from 1998 to 2000 were excluded from cost analyses as no NIS guidelines exist to convert charges to costs.

Multivariable regression modeling was used to estimate the trend in admission-level hospital costs by payer source, adjusted for patient-level confounders and hospital characteristics. Confounding variables were identified through a directed acyclic graph (DAG) and included individual patient variables (sex, age, race, income, E-code category, diagnosis category, and died during hospitalization) and hospital-level variables (location/teaching status: rural, urban non-teaching, and urban teaching; region: Northeast, South, Midwest, and West). Variables such as hospital length of stay and severity were not included because they were identified in the DAG as intermediates.

Generalized linear mixed models (GLMMs) based on the gamma distribution with a log link function and random hospital-level intercepts and slopes best fit the data. The GLMMs were fit via adaptive quadrature to obtain maximum likelihood estimates. NIS sampling weights were incorporated in the GLMMs to obtain nationally representative estimates. Trend weights were used in place of the standard discharge weights for 2001–2011 to accommodate the 2012 re-designed NIS sampling scheme, which allowed for the analysis of a time trend. These models were fit to incorporate both the admissions-level and hospital-level variables. Admissions data were aggregated to the hospital level by summing hospital costs for each unique combination of the aforementioned confounding variables. After fitting the GLMM to the aggregated hospital-level data, national yearly total cost estimates by payer source were obtained by summing the hospital-specific predicted cost. All reported *p* values from GLMMs were obtained by using infinite denominator degrees of freedom. Hypothesis tests were two-sided and used an a priori 0.05 level of significance.

Analyses were conducted using SAS (version 9.4, SAS Institute Inc., Cary, NC, USA). GLMM-based cost estimates were adjusted for quantitative and qualitative covariates by using observed means and relative frequencies, respectively.

Due to missing data in the study for variables of interest (race: 23%; hospital costs: 9.5%; died, sex, location, payer, and income: all <1%), multiple imputation (MI) models were explored using the R (version 3.4.4, Auckland, New Zealand) package mice (version 2.46.0). MI addresses issues associated with missingness in longitudinal datasets, which biases results and reduces the power of associated findings [25]. Multivariate imputation via chained equations were used to create five imputed datasets. Sensitivity analyses were then conducted and showed the results from single imputation yielded comparable standard errors to those from multiple imputation analyses. Therefore, all results are reported from a single imputation analysis. Based on the successful results of the single imputation method compared to the multiple simulated sets, our use of the single imputed should not impact the results and should increase internal validity.

## 3. Results

### 3.1. National Yearly Rate of Child Abuse and Neglect Hospitalizations

**Figure 1:**
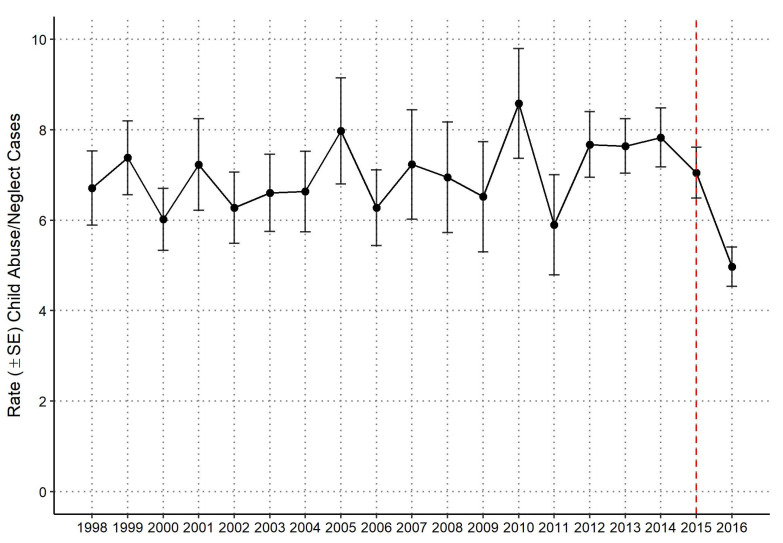
National yearly rate (± standard error) estimates of child abuse and neglect hospitalizations per 100,000 US census population. Dashed vertical red line denotes switch from ICD-9 to ICD-10 standard in Q4 2015.

A weighted total of 101,804 hospital admissions for children with a discharge diagnosis of abuse or neglect were estimated from 1998 through 2016, and 86,766 for 2001–2016 (years for which cost data are available). National yearly estimates of population-based rates of hospitalized child abuse or neglect were calculated using US Census Data with annual population estimates as the rate denominator (Figure 1). The rate of hospitalized child abuse and neglect was 6.7 per 100,000 for the US population in 1998, 8.0 in 2005, and 5.0 in 2016. There was no evidence of a significant time trend in the rate of child abuse or neglect hospitalizations over the study period.

The chi-square test for a significant transition effect from ICD-9-CM to ICD-10-CM found a significant decrease (*p* < 0.0001) in the number of child abuse and neglect cases in the first three quarters (Q1: 1425 [26.0%], Q2: 1560 [28.4%], Q3: 1490 [27.1%]) to the fourth quarter (Q4: 1015 [18.5%]). Thus, any comparisons of results in 2015 and 2016 to previous years should be made with caution, as the ICD-10-CM codes are more specific regarding abuse and adequate training to ensure proper use takes time.

### 3.2. Demographic Characteristics of Children Hospitalized for Child Abuse or Neglect by Sex

Males comprised an estimated 53.0% (Table 1) of all child abuse and neglect hospitalizations from 1998 through 2016. Infants (age < 1) comprised 47.3%, and children age 1–3 accounted for another 24.2%. Female adolescents experienced more abuse or neglect hospitalizations than male adolescents overall and among different age categories. Younger female adolescents (age 10–12) experienced more maltreatment hospitalizations than males (5.8% vs. 4.7%). This gender difference was more pronounced in the 13–15 age group (11.4% vs. 4.0%) and persisted into the teen years of 16–18 (8.9% vs. 2.5%) (*p* < 0.05). White, non-Hispanic children accounted for just over half of admissions, followed by Black children (24.3%) and Hispanic children (16.4%). Children from the lowest income quartile accounted for 34.1% of hospitalizations, whereas children from the highest income quartile accounted for 14.6% of hospitalizations. All distributions and gender comparisons in Table 1 were significant at the *p* = 0.05 level except for income and hospital region.

Most perpetrators (66.4%) had an E-code of Not Otherwise Specified (NOS), other or none. Physical abuse was the most frequent diagnosis (37.6%), followed by NOS, other or none (24.0%) and neglect (13.9%). Children with multiple diagnoses of abuse or neglect accounted for 4.8% of cases.

### 3.3. National Annual Mean and Total Hospital Costs by Payer Source

**Figure 2:**
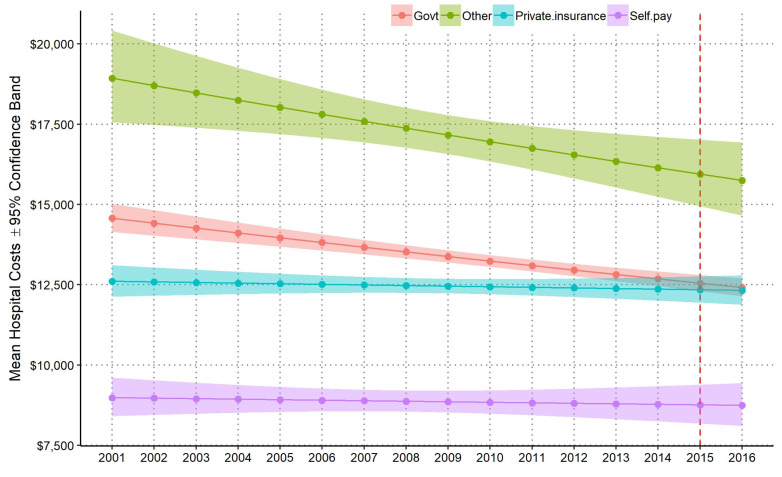
National annual estimates of mean hospital costs per admission (±95% confidence band by payer source from patient-level GLMM). Dashed vertical red line denotes switch from ICD-9 to ICD-10 standard in Q4 2015.

Since 2001, when cost data were available, mean hospital costs per admission generally decreased, but this trend differed by payer source (Figure 2). Results from the GLMM model demonstrated that average costs per hospitalization increased for private insurance and decreased for government and other sources. There were statistically significant differences in mean costs per hospitalization by payer source over the study period based on the GLMM regression analysis (*p* < 0.0001). Controlling for confounding variables, mean hospital costs per admission for government payer sources decreased by −1.1% per year (95% CI [−0.10%, −1.4%], *p* < 0.0001), other payer sources decreased by −0.2% (95% CI [−0.4%, −2.1%], *p* < 0.01) per year, private payer sources slightly decreased by −0.2% (95% CI [−0.06%, 0.3%], *p* = *0*.503) per year, and self-pay payer sources slightly decreased by −0.3% (95% CI [−1.0%, 0.6%], *p* = 0.660) per year. The changes over time between government and private insurance payer sources was also statistically significant (*p* < 0.0001). Mean hospital costs for private insurance sources increased at 0.9% (SE = 0.002) of the mean hospital costs for government sources per year. This trend led to an intersection in 2013; before 2013, the average cost per admission was higher for government than private payers, and after 2013 they were approximately the same. The highest average costs per admission each year were in the “other” payer category and lowest for self-pay.

**Figure 3:**
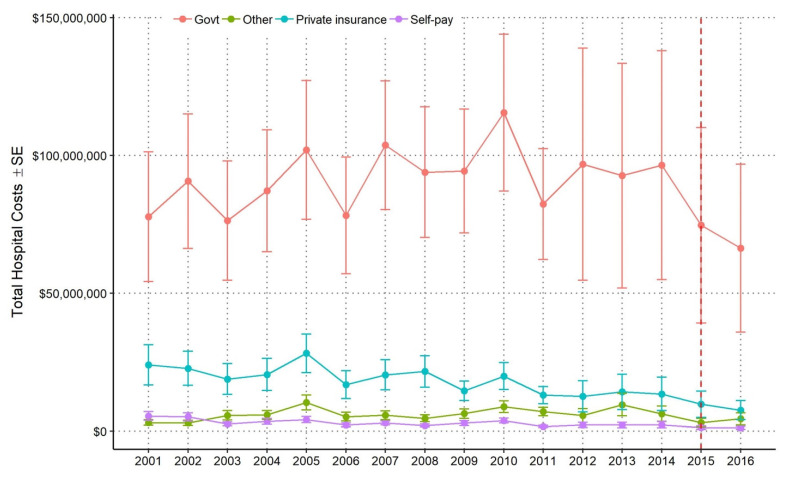
National annual estimates of total hospital costs ± standard error by payer source from hospital-aggregated GLMM. Dashed vertical red line denotes switch from ICD-9 to ICD-10 standard in Q4 2015.

From 2001–2016, the estimated total hospital costs for government payers was $1.4 billion, compared to $420 million for all other payer sources combined. Total costs for all child maltreatment admissions increased for government payers but remained fairly stable over time (Figure 3). Every year since 2001, government sources covered 70% or more of the total hospital costs; although mean hospital costs per admission paid by government sources decreased over time, the total hospital costs paid by government sources increased (Figure 3, Table 2). The increase in total hospital costs covered by government sources was thus driven by an increase in the proportion of admissions paid by government payers over time (Table 2). Total hospital costs paid by government payer sources were substantially larger than the other three payer sources (Table 2). In 2001, government payers paid 70.4% of total hospital costs ($77.7 million), whereas private sources paid 29.6% of total costs ($32.7 million). In 2014, the proportion of payments made to hospitals by government payers increased (70.4% in 2001 to 81.3% in 2016) to a total of $96.5 million, whereas the proportion for all other payer sources decreased (29.6% in 2001 to 18.7% in 2014) to a total of $18.7 million. The decrease in costs in 2015 and 2016 was due to the transition from ICD-9-CM to ICD-10-CM.

## 4. Discussion

This study adds to existing cross-sectional data by examining sixteen-year trends of child abuse and neglect hospitalizations and thirteen-year trends of associated hospital costs. Our results demonstrated no significant change in child abuse and neglect hospitalization rates during the study period. Our findings corroborate results from Farst and colleagues, who reported unchanging rates of hospitalizations due to child abuse injuries between 1997 and 2009 of youth age 0–3 years of age [14]. Our study adds to these findings by examining child abuse and neglect hospitalizations from youth 0–18 years old. Together, these stable rates of maltreatment hospitalizations are alarming given the concerted efforts at the federal level to prevent child abuse and neglect through amendments to the Child Abuse Prevention and Treatment Act (CAPTA) and funding of Community-Based Child Abuse Prevention Programs (CBCAP) [26].

We also investigated the frequency of child maltreatment for a broad range of demographic characteristics. Consistent with previous cross-sectional research on child abuse and neglect hospitalization rates [18,19,20], we found that boys under three years who received Medicaid (government payer) had the highest frequency of child maltreatment hospitalizations across the sixteen years.

Our study also extends Farst’s 12-year analysis of youth ages 0–3 by examining the cost of hospitalizations for youth ages 0–18 as well as payer source [14]. The results from our study demonstrate that government payers cover more than 70% of total hospital costs related to child maltreatment hospitalizations. Our findings complement existing research on the economic burden of child maltreatment. Gelles and Perlman [27] estimated that the U.S. paid $80 billion in 2012 to address short-term and long-term costs associated with child maltreatment, including mental health services and lost wages. The results from this study and existing literature, taken together, demonstrate that child maltreatment remains a persistent and costly problem for society.

Our study has some limitations. Although the National Inpatient Sample is the largest and most comprehensive national database of hospitalizations in the United States, child abuse and neglect hospitalizations are relatively rare, and rates will be less stable and representative than overall hospitalizations. In 2015, the coding structure switched from ICD-9 codes to ICD-10 codes, and rates of child maltreatment hospitalizations examined by quarter show the potential for a coding-driven decrease. ICD-10 codes are more specific for child abuse and include substantiated abuse, potentially changing the types of cases that are included.

Despite these limitations, child maltreatment remains an ongoing public health crisis with substantial personal costs to maltreated children and their families, as well as economic costs to society. According to our findings, there were no significant changes in rates of child abuse and neglect hospitalizations over the sixteen-year study period. Further, the government shoulders the majority of costs for maltreatment hospitalizations, which totaled an estimated $1.4 billion from 2001–2016. Over the period studied, costs per hospitalization increased for government payers but decreased for private payers, indicating an increasing public cost burden. While our study did not examine child abuse and neglect prevention strategies, our findings highlight that rates of child abuse and neglect hospitalizations have not decreased despite existing prevention strategies.

Hospital staff are uniquely situated to identify and prevent child maltreatment. Hospitals serve the entire community and often are the first to interact with vulnerable families. Hospital staff should consider adopting both primary and secondary prevention strategies to reduce child maltreatment hospitalizations and interrupt the relationship between adverse childhood experiences, including child abuse and neglect, and long-term negative health outcomes [28,29,30]. Prevention programs range in delivery cost, yet the payoff is substantial. Demonstrated benefits include improved child wellbeing and parent–child relationships, reduced problem behaviors among children and fewer hospitalizations due to child maltreatment [31,32].

There are several primary child maltreatment prevention programs that hospitals might consider, including the Triple P-Positive Parenting Program and Period of PURPLE Crying model. Triple P-Positive Parenting Program has been implemented across communities and has demonstrated significant reductions in child maltreatment and foster care placements [30]. The Period of PURPLE Crying model, for example, reduced the rate of SBS hospitalizations for infants under ages 1 and 2 by 33% and 35%, respectively [32]. Further, Dias et al. tested a model to prevent SBS, which reduced SBS by 47% among newborns delivered at the hospital [33]. The model cost only $10 per infant and was projected to save more than $21,925 per child per year. These studies indicate the cost-saving benefits of prevention when hospitals invest in primary and secondary prevention. Hospitals also have the opportunity to implement trauma informed care, which can help break the cycle of abuse within families [34,35,36].

Beyond prevention work directly from hospitals. Taking a more holistic view to prevent child maltreatment has benefits for society as well. Prevention programs reduce the overall burden placed on our educational, juvenile justice and medical systems, as well as society at large. The World Health Organization and The International Society for the Prevention of Child Abuse and Neglect [37] have outlined strategies for preventing child maltreatment by the level of influence/intervention and the corresponding developmental stage of the youth. Given the relatively stable trends in child maltreatment reported in this study and the global reports of child maltreatment [1,2,3], child maltreatment poses a complex problem that requires a complex and comprehensive solution. Disrupting the cycle of maltreatment requires that society adjust its approach to maltreatment prevention and allocate the dollars to prevent child maltreatment not just at the parenting level but looking to more societal and community strategies as outlined by WHO. Van Dijken and colleagues [38] contend that there are contextual factors that are at play and need to be addressed from a level of community-based intervention to explain why individual prevention programs that serve families can be effective, but yet we still have high rates of child maltreatment. These contextual factors need to account for the environment and systems that parents raise their children and that children and families participate in, such as school and healthcare systems.

## 5. Conclusions

Child maltreatment remains an ongoing public health crisis with substantial personal costs to maltreated children and economic costs to society. According to our findings, the government shoulders most costs for maltreatment hospitalizations, which totaled an estimated $1.4 billion from 2001–2016. Disrupting the cycle of maltreatment requires that our society adjust its approach to maltreatment prevention and allocate the dollars to prevent child maltreatment. Hospitals should collaborate with community partners to implement effective primary prevention programs, which can reduce child maltreatment, a preventable public health problem, resulting in benefits for children and society.

## Figures and Tables

**Table 1:** Demographic Characteristics by Sex: National Estimates (1998–2016).

	Female	Male	Overall
	No. (%)	No. (%)	No. (%)
Sex	47,811 (47.0)	53,993 (53.0)	101,804 (100)
Age			
<1	19,973 (41.8)	28,141 (52.1)	48,114 (47.3)
1–3	10,575 (22.1)	14,019 (26.0)	24,594 (24.2)
4–6	2687 (5.6)	3177 (5.9)	5864 (5.8)
7–9	2104 (4.4)	2598 (4.8)	4702 (4.6)
10–12	2752 (5.8)	2513 (4.7)	5265 (5.2)
13–15	5457 (11.4)	2172 (4.0)	7629 (7.5)
16–18	4262 (8.9)	1373 (2.5)	5635 (5.5)
Race/Ethnicity			
Asian or Pacific Islander	621 (1.3)	672 (1.2)	1293 (1.3)
Black	11,676 (24.4)	13,109 (24.3)	24,784 (24.3)
Hispanic	8102 (16.9)	8582 (15.9)	16,684 (16.4)
Other	2594 (5.4)	2722 (5.0)	5316 (5.2)
Native American	587 (1.2)	467 (0.9)	1054 (1.0)
White	24,231 (50.7)	28,442 (52.7)	52,673 (51.7)
Income Quartile based on ZIP Code			
Quartile 1	16,417 (34.3)	18,279 (33.9)	34,696 (34.1)
Quartile 2	13,826 (28.9)	15,587 (28.9)	29,413 (28.9)
Quartile 3	10,567 (22.1)	12,308 (22.8)	22,875 (22.5)
Quartile 4	7000 (14.6)	7819 (14.5)	14,819 (14.6)
Type of Maltreatment (Diagnosis)			
Multiple	2675 (5.6)	2258 (4.2)	4933 (4.8)
Neglect	6150 (12.9)	7996 (14.8)	14,147 (13.9)
Not otherwise specified, other, none	11,314 (23.7)	13,123 (24.3)	24,437 (24.0)
Physical abuse	16,149 (33.8)	22,164 (41.0)	38,313 (37.6)
Psychological abuse	547 (1.1)	376 (0.7)	923 (0.9)
Shaken baby syndrome	4409 (9.2)	6738 (12.5)	11,147 (10.9)
Sexual abuse	6566 (13.7)	1338 (2.5)	7904 (7.8)
Died			
No	46,163 (96.6)	51,702 (95.8)	97,865 (96.1)
Yes	1648 (3.4)	2291 (4.2)	3939 (3.9)
Perpetrator (E-Code)			
Father, step-father, boyfriend	7256 (15.2)	8636 (16.0)	15,891 (15.6)
Mother, step-mother, girlfriend	4605 (9.6)	5490 (10.2)	10,095 (9.9)
Multiple	986 (2.1)	917 (1.7)	1903 (1.9)
Non-relative	968 (2.0)	1219 (2.3)	2186 (2.1)
Not otherwise specified, other, none	31,579 (66.0)	36,053 (66.8)	67,631 (66.4)
Other relative	2418 (5.1)	1679 (3.1)	4097 (4.0)
Hospital Location: Teaching Status			
Rural	3479 (7.3)	3651 (6.8)	7131 (7.0)
Urban: Non-teaching	6844 (14.3)	6739 (12.5)	13,583 (13.3)
Urban: Teaching	37,487 (78.4)	43,603 (80.8)	81,090 (79.7)
Hospital Region			
Midwest	12,573 (26.3)	14,314 (26.5)	26,887 (26.4)
Northeast	7781 (16.3)	8021 (14.9)	15,802 (15.5)
South	19,015 (39.8)	21,807 (40.4)	40,823 (40.1)
West	8441 (17.7)	9852 (18.2)	18,293 (18.0)

All overall distributions and gender comparisons were significant at the *p* = 0.05 level.

**Table 2:** National annual estimates of total and percentage of hospital costs paid by government payer sources compared to all other payer sources from hospital-aggregated GLMM.

Year	Government		All Other Payer Sources		Total Costs
	Costs	% of Total Costs	Costs	% of Total Costs	
2001	77,700,000	70.4	32,700,000	29.6	110,000,000
2002	90,600,000	74.6	30,900,000	25.4	122,000,000
2003	76,300,000	73.7	27,300,000	26.3	104,000,000
2004	87,200,000	74.4	30,000,000	25.6	117,000,000
2005	102,000,000	70.4	42,800,000	29.6	145,000,000
2006	78,200,000	76.3	24,300,000	23.7	103,000,000
2007	104,000,000	78.1	29,200,000	21.9	133,000,000
2008	93,900,000	76.8	28,400,000	23.2	122,000,000
2009	94,400,000	79.8	23,900,000	20.2	118,000,000
2010	115,000,000	78.0	32,600,000	22.0	148,000,000
2011	82,300,000	79.0	21,800,000	21.0	104,000,000
2012	96,800,000	82.5	20,600,000	17.5	117,000,000
2013	92,600,000	78.0	26,100,000	22.0	119,000,000
2014	96,500,000	81.3	22,100,000	18.7	119,000,000
2015	74,700,000	84.0	14,200,000	16.0	88,800,000
2016	66,400,000	83.3	13,300,000	16.7	79,700,000
Total	1,430,000,000	77.3	420,000,000	22.7	1,850,000,000

## Data Availability

The National Inpatient Sample (NIS) is a publicly available dataset. Information can be found at https://www.hcup-us.ahrq.gov/db/nation/nis/nisdbdocumentation.jsp.
